# Equitable partnerships in research to achieve the Sustainable Development Goals: pathways for nursing

**DOI:** 10.1590/0034-7167-2024-0183

**Published:** 2025-06-20

**Authors:** Álvaro Francisco Lopes de Sousa, Emerson Lucas Silva Camargo, Carla Aparecida Arena Ventura, Isabel Amelia Costa Mendes

**Affiliations:** I Hospital Sírio-Libanês, Instituto de Ensino e Pesquisa. São Paulo, São Paulo, Brazil; II Universidade NOVA de Lisboa, Escola Nacional de Saúde Pública NOVA. Lisboa, Portugal; III Universidade de São Paulo. Ribeirão Preto, São Paulo, Brazil

**Keywords:** Sustainable Development Goals, Agenda 2030, Leadership, Nurse, Health Priorities, Objetivos de Desarrollo Sostenible, Agenda 2030, Liderazgo, Enfermero, Prioridades de Salud

## Abstract

**Objective:**

To discuss the importance of fair and equitable partnerships in research to achieve the Sustainable Development Goals (SDGs), and implications for nursing practice.

**Methods:**

This is a theoretical-reflexive study, with a systematic search for articles and reflective analyses to identify central themes and build critical arguments. This is a theoretical-reflexive study, with a systematic search for articles and reflective analyses to identify central themes and build critical arguments.

**Results:**

Equitable research partnerships strengthen local capacity, address global health inequalities, and promote the inclusion of diverse perspectives. They contribute significantly to the achievement of the SDGs, especially in strengthening research infrastructure in low- and middle-income countries, and in promoting evidence-based policies for health equity.

**Conclusion:**

Establishing and maintaining fair and equitable partnerships is crucial to addressing complex global challenges and achieving the SDGs by 2030. Nursing plays a vital role in this process, advocating for equity, leading community-based research, and contributing to evidence-based policies.

## RESUMEN

**Objetivo:**

Discutir la importancia de las alianzas justas y equitativas en la investigación para alcanzar los Objetivos de Desarrollo Sostenible (ODS), y las implicaciones para las prácticas de enfermería.

**Métodos:**

estudio teórico-reflexivo, con búsqueda sistemática de artículos y análisis reflexivos para identificar temas centrales y construir argumentos críticos.

**Resultados:**

Las asociaciones de investigación equitativas fortalecen la capacidad local, abordan las desigualdades en materia de salud mundial y promueven la inclusión de perspectivas diversas. Contribuyen significativamente al logro de los ODS, especialmente en el fortalecimiento de la infraestructura de investigación en los países de ingresos bajos y medios y en la promoción de políticas basadas en evidencia para la equidad en salud.

**Conclusión:**

Establecer y mantener alianzas justas y equitativas es crucial para abordar desafíos globales complejos y alcanzar los ODS para 2030. La enfermería desempeña un papel vital en este proceso al abogar por la equidad, liderar la investigación comunitaria y contribuir a políticas basadas en evidencia.

## INTRODUCTION

The Sustainable Development Goals (SDGs) are an international set of targets designed to promote development and act as a “global call” for concerted action by the international community to eradicate poverty, protect the environment and climate, and ensure that people everywhere can enjoy peace and prosperity. Aimed at being achieved by the year 2030, the SDGs consist of 17 goals and 169 interconnected targets

These goals must seek to achieve a balance between environmental, social and economic sustainability and, because they are interconnected, it is recognized that all areas must advance and that decisions taken in one area will have an effect on results in other areas. More recently, the COVID-19 epidemic appears to have exacerbated, exposed and widened global inequality; however, the 2030 Agenda and its SDGs remain a crucial foundation for the recovery and reconstruction of a better society, ensuring that “no one is left behind”^(1)^.

Given the scope and magnitude of these goals, achieving them will require efforts by individual nations as well as by groups of nations, who must act locally while also thinking globally and taking into account their unique circumstances. Research collaborations are one of the most promising approaches to achieving the SDGs. Partnerships between diverse organizations are necessary for international growth, as is the combination of diverse professions and expertise through joint ventures with representatives of the public, commercial, and government sectors ^(2)^.

Historically, inequality has defined research partnerships, with developed nations having much easier access to financial and technological resources, enabling them to conduct cutting-edge research, while developing nations face issues such as inadequate funding, poor infrastructure, and brain drain. Limited access to information is the first step towards inequality. Professionals and researchers in the Global South cannot afford the high costs of open access journals and books, and academic and service organizations often do not offer this benefit. The ability of developing nations to apply, profit from, and even contribute to research is weakened by this discrepancy^(3)^.

In this context, Brazil is experiencing a particular case of increasing disorganization and lack of funding for science and technology, partly a reflection of poor infrastructure, discontinuity, and inadequate funding and support for research. This situation over the years creates a vicious cycle in which the country’s scientific and technical capabilities cannot fully develop due to lack of funding and resources.

In this context, “fair and equitable research partnerships” emerge as research partnerships in which there is mutual trust, participation and respect, as well as reciprocal benefits and equal value given to the contribution of each partner at all stages of the research process. Such initiatives are essential to achieving the SDGs. Brazil is an important country in the global South and faces the challenge of ensuring universal, high-quality health for all, capable of promoting well-being in a variety of sociocultural contexts. Nursing is a very important “key player” in this scenario and the SDGs offer a comprehensive framework to guide such efforts.

## OBJECTIVE

Discuss the importance of fair and equitable partnerships in research to achieve the SDGs, as well as the implications for nursing practices.

## METHODS

This is a theoretical-reflective study, which used an exploratory and interpretative method to highlight the importance of fair and equitable research collaborations in the pursuit of the SDGs. The study was based on a systematic evaluation of official documents together with the scientific literature, combined with reflective analysis based on the experiences of researchers working in international partnerships and research.

### The following steps were followed in this study:

1. Literature Review: Using keywords such as research collaborations, equality, justice, nursing and SDGs, a thorough search was carried out in the *PubMeddatabases, Scopus* and *Web of Science.* Manuscripts published in all languages since 2015 were the main focus of the article selection process, which also targeted publications on theories, models and practical applications of equitable research collaborations.
2. Reflective Analysis: Following the literature review, the authors held discussion sessions to review the data collected, their own experiences working on collaborative research projects, and the state of nursing in relation to the SDGs. Reflective Thematic Analysis developed by Virginia Braun and Victoria Clarke was used as an approach to facilitate the identification of important themes and the development of persuasive arguments about fair and equitable research collaborations.
3. Knowledge Synthesis: Using content analysis as a basis, the authors synthesized knowledge from the literature review and reflective analysis, focusing on practical implications for improving nursing research collaborations.
4. Analytical Technique: A qualitative technique was used to analyze the selected resources, and content analysis was used to identify important themes and patterns. Through this technique, the authors were able to investigate the theoretical and practical aspects of equitable research collaborations and how they contribute to the goals of the nursing field.

## RESULTS

Why is it important to seek research partnerships that are fair and equitable?

The current model of research cooperation places excessive priority on the collaboration of Teaching and Research Institutions from geographically distinct locations, whether within the same country (in the case of Brazil, there is a strong incentive for collaboration with the North and Central-West regions) or between institutions from the Global North and South, for example.

However, little attention has been given to interdisciplinary collaboration that encompasses sectors beyond academia, such as civil society, government, and the private sector. Focusing research efforts on fair and equitable partnerships is based on the recognition that different individuals and institutions, regardless of their geographic location, bring with them a diversity of relationships, knowledge, skills, and perspectives to the processes of research development and implementation of results.

By working in partnership, many stakeholders can collectively develop a deeper understanding of global challenges and possible responses to them, as well as new processes, practices and research products. They can generate insights and evidence to improve development practices and policies, contribute to the eradication of poverty and promote more just and equitable societies. However, for these partnerships to achieve these aspirations, it is necessary to consider the meaning of justice and equity and then focus on the purpose of translating these ideas into practice, defining the means and human resources to be involved, leading intergroup and inter-institutional processes and negotiations in order to ensure their implementation. At the same time, it is necessary to advocate for investments in interested entities to promote consecutive research projects, as well as to understand the results of the changes implemented in the relevant sectors referred to by the SDGs^(4)^.

### Key challenges for fair and equitable research partnerships

The COVID-19 pandemic has exposed the challenges facing global health and highlighted the urgent need for cross-border cooperation and research alliances. The global crisis caused by SARS-CoV-2 has highlighted the essential need for collaborative work, as well as the establishment of effective research partnerships to accelerate studies and vaccine development in record time, enabling the use of these innovations on a global scale. This scenario has reinforced the idea that research partnerships are not only beneficial, but fundamental to addressing critical and multifaceted challenges^(5)^.

Despite growing recognition of the importance of research collaborations, significant obstacles remain, particularly in creating and maintaining fair and equitable partnerships. Notable disparities are observed when teams from different nations collaborate with each other, with those from middle-income countries facing significant obstacles, resulting in an imbalance of power and resources, while teams from high-income countries often have an abundance of resources and a much more robust infrastructure.

These disparities occur in several areas related to collaborative research. Significant discrepancies can be reflected in issues such as research design and planning, study execution, data analysis and interpretation, and, most importantly, how benefits, costs, and outcomes are distributed and incorporated into day-to-day operations. This unequal distribution raises ethical issues and also compromises the effectiveness of collaborations, potentially perpetuating cycles of dependency and impeding the development of sustainable research infrastructures in low- and middle-income nations.

Recognizing these disparities and challenges is a crucial step, necessary but insufficient. To progress toward genuinely fair and equitable collaborations, it is crucial to adopt methods that prioritize collaborative knowledge development, fair allocation of resources, transparency in decision-making, and parity in authorship and recognition. This requires not only a theoretical commitment to fairness and equity, but also the practical application of guidelines and practices that promote these ideals throughout the collaborative research process.

Building and maintaining equitable partnerships requires ongoing reflection and a commitment to continuous improvement, ensuring that all voices are heard and valued, and that the benefits of research are shared fairly and equitably. It is a complex challenge, but one that is crucial for advancing research to be not only innovative, but also responsible, inclusive and, moreover, for its progress to transcend the researchers involved and involve their institutions locally and, through them and their leadership and influence, other national, regional and global instances are impacted, in a dynamic and transformative movement of reality.

Constant evaluation and dedication to continuous development are necessary to create and sustain fair and equitable collaborations, ensuring that all opinions are heard, that no one is left behind, and that everyone’s culture and aspirations are respected, ensuring that the benefits of research are distributed equally. Although it may seem like a daunting task, it is essential that research is innovative, responsible, inclusive and that its advancement goes beyond individual researchers and their institutions locally, allowing other national, regional and global bodies to be impacted in a dynamic and transformative movement of reality.

Fair and equitable research collaborations face many complex obstacles, especially when considering relationships between institutions in the Global North and South. Research institutes in wealthy countries often hold a disproportionate amount of resources in global health research collaborations. Brazil’s experience during the COVID-19 pandemic amply demonstrates this dynamic, especially given the country’s financial constraints on research at this crucial time^(5)^.

Brazil and several other middle-income nations have seen significant reductions in their research budgets during the pandemic. This has had a direct impact on the country’s ability to address the health emergency, conduct independent research on the virus, treatments and vaccines, and contribute independently to the global understanding of the pandemic. This disparity may perpetuate a neocolonialist dynamic already observed in other contexts in which wealthier nations set the research agenda, ignoring the voice, representation and independence of institutions in developing countries. Therefore, to advance equity, it is imperative that the international community adopts concrete anti-racist and anti-colonial measures. Researchers and staff from developing nations must be allowed to play a more proactive and decisive role in collaborations, from identifying local problems, goals and solutions to negotiating the terms of international partnerships in which they are involved.

### The impact of fair and equitable partnerships on the SDGs

Fair and equitable research partnerships have a profound and multifaceted impact on achieving the SDGs set for 2030. Collaboration between high-income countries and low- and middle-income countries on the basis of fairness and equity is not only an ethical imperative, but also an effective strategy to accelerate global progress towards these goals. By analyzing the perspectives and conclusions presented in the literature, we can gain a better understanding of the influence of such partnerships on the SDGs^(6)^.

- Strengthening Local Research Capacity (SDG 9 – Industry, Innovation and Infrastructure): Fair and equitable partnerships contribute to the development of robust research infrastructures in developing countries, fostering local innovation and enabling these countries to not only participate in, but also lead research efforts that address their specific needs. This not only improves local research capacity, but also promotes self-reliance and reduces dependence on external interventions.
- Incorporation of Multiple Perspectives (SDG 5 - Gender Equality, SDG 10 - Reduced Inequalities): Equitable partnerships ensure that a wide range of voices and perspectives are included in research, which is vital to addressing gender and inequality issues. Including marginalized and underrepresented groups in research processes ensures that their needs and challenges are adequately understood and addressed.
- Promoting Quality Education (SDG 4): By engaging institutions in developing countries in equitable partnerships, educational and training capacity in these regions is enhanced. This leads to an improvement in the quality of education through the establishment of training and capacity-building programmes relevant to local needs and aligned with global best practices.
- Addressing Health Inequalities (SDG 3 - Good Health and Well-being): Fair and equitable partnerships in global health are crucial to addressing health inequalities. By collaborating on equal terms, countries and institutions can develop and implement culturally appropriate, affordable and accessible health solutions for all populations, especially the most vulnerable.
- Accelerating Implementation and Impact (SDG 17 - Partnerships for the Goals): Fair and equitable partnerships are examples of SDG 17, which emphasizes the importance of partnerships in achieving the SDGs. They illustrate how collaboration based on mutual respect, equality and benefit-sharing can accelerate progress towards all the goals.

### Fair and equitable partnerships in research are the way to 2030

Fair and equitable research partnerships play a critical role in achieving the 2030 Agenda for Sustainable Development. Because of their interconnectedness, they require collaboration across countries, sectors and disciplines. Research partnerships, when conducted in a fair and equitable manner, can accelerate this process in several significant ways.

Fair and equitable partnerships facilitate knowledge co-creation, combining local and global expertise to address sensitive and challenging issues. The interdisciplinary and multicultural approach provided is crucial to understanding and addressing the multifaceted challenges that the SDGs seek to address. Knowledge co-creation also ensures that solutions are culturally appropriate, socially acceptable and technically feasible^(7)^.

Partnerships can enhance institutional and individual capacities in low- and middle-income countries. By sharing knowledge, resources and technologies, institutions in high-income countries can contribute to the development of sustainable research infrastructures adapted to the specific realities and needs of developing countries. This not only supports local research, but also contributes to the development of skilled professionals and robust institutions, which are essential for sustainable progress^(8)^.

Equitable partnerships directly address global inequalities by seeking to promote a fairer balance of power, resources and recognition. In doing so, they contribute to achieving the SDGs related to reducing inequalities (SDG 10) and promoting peaceful and inclusive societies for sustainable development (SDG 16).

Through partnerships that value local perspectives, global goals and strategies can be aligned with local needs and priorities. This decentralized approach is crucial to the success of the SDGs, as it recognizes that effective solutions must be rooted in the local context.

Equitable partnerships can support research and development of solutions that promote environmental sustainability (SDGs 13, 14 and 15) and ensure social and economic well-being (SDGs 1, 2, 3, etc.). By combining different types of knowledge and resources, these partnerships can innovate in areas such as health, education, sustainable agriculture and clean energy.

Fair and equitable research partnerships can further promote peace and justice by addressing the underlying structural roots of inequalities and promoting inclusive and just societies, as outlined in SDG 16.

### The Role of Nursing in Fair and Equitable Research Partnerships

Nursing, both as a science and as a practice, plays a crucial role in fair and equitable research partnerships, especially within the framework of the SDGs. The profession’s holistic, patient-centered orientation, coupled with its commitment to culturally competent care, places it in a unique position to influence and lead research partnerships aimed at achieving the SDGs^(9)^. There are several ways in which nursing can position itself and contribute in a meaningful way:

- Advocacy for Health Equity: Nurses, as a leadership position, can advocate for health equity by working to ensure that the voices of underrepresented communities are heard in research partnerships. Nurses are often on the front lines of care, which puts them in a unique position to understand the needs of communities and advocate for the inclusion of these perspectives in the research agenda.
- Community-based research: Nurses can lead and participate in community-based research, ensuring that research questions are relevant to local needs and that research findings are translated into practices that directly benefit the communities served.
- Training and Education: Nursing can play a crucial role in empowerment and education, both locally and globally. Nursing education programs can include components that emphasize the importance of fair and equitable research partnerships, thus preparing the next generation of nurses to be effective and conscientious collaborators in global research.
- Leadership in Interdisciplinarity: Due to its interdisciplinary nature and central positioning in health systems, nursing is well positioned to lead or participate in multidisciplinary research teams. This can promote a more holistic approach to research, ensuring that multiple perspectives are included and that the results are more robust and applicable, as long as it claims a seat at the decision-making table and brings its expertise to the table by offering suggestions, plans and implementation strategies.
- Advocacy for Evidence-Based Policies: Nurses, as advocates for evidence-based policies, can use research findings to influence and shape health policies that promote equity, justice, and sustainable development. To be able to do this, nurses need to gain access to policy-making spaces, be up-to-date on the evidence produced by nursing, and then lead and present informed proposals that influence policymaking.
- Through global partnerships, nurses can collaborate with colleagues in different regions to share knowledge, resources and best practices. This not only improves local capacities but also fosters a deeper understanding of the social determinants of health in diverse cultural and geographic contexts.

By assuming the roles of leaders, educators, researchers, and advocates, nurses can play a crucial role in ensuring that research partnerships are fair, equitable, and aligned with the goals designed to promote global health and sustainable development. Nursing, with its unwavering commitment to caring, empathy, human rights affirmation, and social justice, has a significant role to play in achieving these goals through strong and collaborative research partnerships^(10)^.

## FINAL CONSIDERATIONS

Our study highlights the critical need for fair and equitable research partnerships on the path to achieving the Sustainable Development Goals (SDGs) by 2030. By recognizing the diversity of perspectives, expertise, and resources that different institutions and individuals bring to the research process, we can foster a more holistic and inclusive approach to addressing global health and development challenges. By strengthening local capacities, incorporating other perspectives, and promoting health equity, research partnerships can play a crucial role in transforming our societies towards a more just and sustainable future.

Furthermore, this study highlights the crucial role of nursing as a protagonist in promoting equitable research partnerships and in the pursuit of achieving the SDGs. The nursing profession, with its holistic and patient-centered approach, is well positioned to lead community research initiatives, advocate for evidence-based policies, and strengthen local health capacities. By actively integrating nursing into research partnerships, we can ensure that solutions are culturally sensitive, socially equitable, and clinically effective, thereby driving progress towards the SDGs.
