# Supplementary material for: Equitable partnerships in research to achieve the Sustainable Development Goals: pathways for nursing
Source: Rev Bras Enferm. 2025 Jun 20;78(Suppl 1):e20240183. doi: 10.1590/0034-7167-2024-0183 (PMC12185115; doi:10.1590/0034-7167-2024-0183)
Supplement: Translation

# Translation: Parcerias equitativas em pesquisa para alcançar os Objetivos de Desenvolvimento Sustentável: caminhos para a enfermagem

**DOI:** 10.1590/0034-7167-2024-0183pt

**Authors:** Álvaro Francisco Lopes de Sousa, Emerson Lucas Silva Camargo, Carla Aparecida Arena Ventura, Isabel Amelia Costa Mendes

**Affiliations:** I Hospital Sírio-Libanês, Instituto de Ensino e Pesquisa. São Paulo, São Paulo, Brasil; II Universidade NOVA de Lisboa, Escola Nacional de Saúde Pública NOVA. Lisboa, Portugal; III Universidade de São Paulo. Ribeirão Preto, São Paulo, Brasil

**Keywords:** Objetivos de Desenvolvimento Sustentável, Agenda 2030, Liderança, Enfermeiro, Prioridades em Saúde

## Abstract

**Objetivo:**

Discutir a importância das parcerias justas e equitativas em pesquisa para alcançar os Objetivos de Desenvolvimento Sustentáveis (ODS), e implicações para as práticas de enfermagem

**Métodos:**

estudo teórico-reflexivo, com busca sistemática de artigos e análises reflexivas para identificar temas centrais e construir argumentos críticos.

**Resultados:**

As parcerias de pesquisa equitativas fortalecem a capacidade local, abordam desigualdades globais de saúde e promovem a inclusão de diversas perspectivas. Elas contribuem significativamente para a consecução dos ODS, especialmente no fortalecimento da infraestrutura de pesquisa em países de renda média e baixa, e na promoção de políticas baseadas em evidências para a equidade em saúde.

**Conclusão:**

Estabelecer e manter parcerias justas e equitativas é crucial para enfrentar desafios globais complexos e alcançar os ODS até 2030. A enfermagem desempenha um papel vital nesse processo, advogando pela equidade, liderando pesquisas comunitárias e contribuindo para políticas baseadas em evidências.

## INTRODUÇÃO

Os Objetivos de Desenvolvimento Sustentável (ODS) são um conjunto internacional de metas projetadas para promover o desenvolvimento e agir como um “apelo global” para ação conjunta da comunidade internacional para erradicar a pobreza, proteger o meio ambiente e o clima, e garantir que as pessoas em todos os lugares possam desfrutar de paz e prosperidade. Com o objetivo de serem alcançados até o ano de 2030, os ODS são compostos por 17 metas e 169 objetivos interligados^(1)^.

Esses objetivos devem buscar alcançar um equilíbrio entre sustentabilidade ambiental, social e econômica e por estarem interconectados reconhece-se que todas as áreas devem avançar e que as decisões tomadas em uma área terão efeito nos resultados nas outras áreas. Mais recentemente, a epidemia de COVID-19 parece ter exacerbado, exposto e ampliado a desigualdade global; no entanto, a Agenda 2030 e seus ODS permanecem como uma base crucial para a recuperação e reconstrução de uma sociedade melhor, garantindo que “ninguém seja deixado para trás”^(1)^.

Considerando o alcance e a magnitude desses objetivos, alcançá-los exigirá esforços de cada nação isoladamente, bem como do grupo de nações, que devem agir localmente enquanto também pensam globalmente e levam em consideração suas circunstâncias únicas. As colaborações em pesquisa são uma das abordagens mais promissoras para alcançar os ODS. Parcerias entre diversas organizações são necessárias para o crescimento internacional, assim como a combinação de diversas profissões e especialidades por meio de empreendimentos conjuntos com representantes dos setores público, comercial e governamental^(2)^

Historicamente, a desigualdade define as parcerias em pesquisa, com as nações desenvolvidas com acesso muito mais fácil a recursos financeiros e tecnológicos, permitindo-lhes realizar pesquisas de ponta, enquanto as nações em desenvolvimento enfrentam questões como financiamento inadequado, infraestrutura precária e fuga de cérebros. O acesso limitado à informação é o primeiro passo para a desigualdade. Profissionais e pesquisadores do Sul -Global não podem pagar os altos custos de periódicos de acesso livre e livros, e organizações acadêmicas e de serviço geralmente não oferecem esse benefício. A capacidade das nações em desenvolvimento de aplicar, lucrar e até contribuir para a pesquisa é enfraquecida por essa discrepância^(3)^.

Nesse contexto, o Brasil vive um caso em particular com crescente desorganização e falta de financiamento da ciência e tecnologia, em parte, reflexo da má infraestrutura, descontinuidade, financiamento e apoio inadequados à pesquisa. Essa situação ao longo de anos cria um ciclo vicioso no qual as habilidades científicas e técnicas do país não conseguem crescer plenamente devido à falta de financiamento e recursos.

Nesse contexto, as “parcerias justas e equitativas em pesquisa” surgem como parcerias em pesquisa nas quais há confiança, participação e respeito mútuos, bem como benefícios recíprocos e de igual valor dado à contribuição de cada parceiro em todas as etapas do processo de pesquisa. Tais iniciativas são essenciais para o alcance dos ODS. O Brasil é um importante país do Sul global e enfrenta um desafiante de garantia de saúde para todos com acesso universal, de alta qualidade e capaz de incentivar o bem-estar em uma variedade de contextos socioculturais. A Enfermagem, é um “jogador-chave” importantíssimo nesse cenário e os ODS oferecem um quadro abrangente para orientar tais esforços.

## OBJETIVO

Discutir a importância das parcerias justas e equitativas em pesquisa para alcançar os ODS, bem como as implicações para as práticas de enfermagem.

## MÉTODOS

Trata-se de um estudo teórico-reflexivo, que utilizou-se de método exploratório e interpretativo para destacar a importância de colaborações de pesquisa justas e equitativas na busca pelos ODS. O estudo teve como base uma avaliação sistemática de documentos oficiais em conjunto com a literatura cientifica, aliado a análise reflexiva baseada nas experiências dos pesquisadores trabalhando em parcerias internacionais e pesquisas.

### As seguintes etapas foram seguidas neste estudo:

1. Revisão da Literatura: Utilizando palavras-chave como colaborações de pesquisa, igualdade, justiça, enfermagem e ODS, foi realizada uma busca minuciosa nas bases de dados *PubMed, Scopus* e *Web of Science.* Os manuscritos publicados em todos os idiomas a partir de 2015 foram o foco principal do processo de seleção do artigo, que também visou publicações sobre teorias, modelos e aplicações práticas de colaborações de pesquisa equitativas.
2. Análise Reflexiva: Após a revisão da literatura, os autores realizaram sessões de discussão para revisar os dados coletados, suas próprias experiências trabalhando em projetos de pesquisa colaborativa e o estado da enfermagem em relação aos ODS. A Análise Temática Reflexiva desenvolvida por Virginia Braun e Victoria Clarke foi utilizada como abordagem para facilitar a identificação de temas importantes e o desenvolvimento de argumentos persuasivos sobre colaborações de pesquisa justas e equitativas.
3. Síntese do Conhecimento: Utilizando a análise de conteúdo como base, os autores sintetizaram o conhecimento da revisão da literatura e da análise reflexiva, concentrando-se nas implicações práticas para melhorar as colaborações de pesquisa em enfermagem.
4. Técnica Analítica: Uma técnica qualitativa foi utilizada para analisar os recursos selecionados, e a análise de conteúdo foi usada para identificar temas e padrões importantes. Por meio dessa técnica, os autores puderam investigar os aspectos teóricos e práticos das colaborações de pesquisa equitativas e como elas contribuem para os objetivos do campo da enfermagem.

## RESULTADOS

Por que é importante buscar parcerias de pesquisa que sejam justas e equitativas?

O modelo atual de cooperação em pesquisa tem priorização excessiva entre a colaboração de Instituições de Ensino e Pesquisa de locais geograficamente distintos, seja dentro do mesmo país (no caso do Brasil, há um forte estímulo para a colaboração com as regiões Norte e Centro-Oeste) ou entre instituições do Norte e Sul Global, por exemplo.

No entanto, pouca atenção tem sido dada à colaboração focada na interdisciplinaridade que contemple setores além da academia, como a sociedade civil, governo e setor privado. Concentrar esforços de pesquisa em parcerias justas e equitativas parte do reconhecimento de que diferentes indivíduos e instituições, independente da sua área geográfica, trazem consigo uma diversidade de relacionamentos, conhecimentos, habilidades e perspectivas para os processos de desenvolvimento da pesquisa e implementação dos resultados.

Ao trabalhar em parceria, muitos interessados podem desenvolver coletivamente uma compreensão mais profunda dos desafios globais e das possíveis respostas a eles, assim como novos processos, práticas e produtos de pesquisa. Eles podem gerar insights e evidências para melhorar práticas e políticas de desenvolvimento, contribuir para a erradicação da pobreza e promover sociedades mais justas e equitativas. No entanto, para que essas parcerias alcancem essas aspirações, é necessário considerar o significado de justiça e equidade e, em seguida, focar no propósito de traduzir essas ideias para a prática, definindo os meios e os recursos humanos a serem envolvidos, liderando processos intergrupais e interinstitucionais e negociações, a fim de garantir sua implementação. Simultaneamente, pleitear investimentos em entidades interessadas em promover projetos de pesquisa consecutivos, bem como compreender os resultados das mudanças implementadas nos setores relevantes referidos pelos ODS^(4)^.

### Principais desafios para parcerias de pesquisa justas e equitativas

A pandemia de COVID-19 expôs os problemas com a saúde global e destacou a necessidade urgente de cooperação transfronteiriça e alianças de pesquisa. A crise global causada pelo SARS-CoV-2, destacou a necessidade essencial do trabalho colaborativo, bem como do estabelecimento de parcerias de pesquisa eficazes para aceleração dos estudos e desenvolvimento de vacinas em tempo recorde, permitindo o uso destas inovações em escala global. Este cenário reforçou a ideia de que as parcerias de pesquisa não são apenas benéficas, mas fundamentais para enfrentar desafios críticos e multifacetados^(5)^.

Apesar do reconhecimento crescente da importância das colaborações em pesquisa, persistem obstáculos significativos, especialmente na criação e manutenção de parcerias justas e equitativas. Disparidades notáveis são observadas quando equipes de diferentes nações colaboram entre si, com aquelas provenientes de países de classe média e enfrentando grandes obstáculos, resultando em desequilíbrio de poder e recursos, enquanto equipes de países de alta renda frequentemente têm uma abundância de recursos e uma infraestrutura bem mais robusta.

Essas disparidades ocorrem em várias áreas relacionadas à pesquisa colaborativa. Discrepâncias significativas podem ser refletidas em questões como desenho e planejamento de pesquisa, execução de estudos, análise e interpretação de dados e, mais importante, como os benefícios, despesas e resultados são distribuídos e incorporados no dia-a-dia. Essa distribuição desigual gera questões éticas e também compromete a eficácia das colaborações, potencialmente perpetuando ciclos de dependência e impedindo o desenvolvimento de infraestruturas de pesquisa sustentáveis em nações de média e baixa renda.

O reconhecimento dessas disparidades e desafios é um passo crucial, necessário, mas insuficiente. Para progredir em direção a colaborações genuinamente justas e equitativas, é crucial adotar métodos que priorizem o desenvolvimento colaborativo do conhecimento, alocação justa de recursos, transparência na tomada de decisões e paridade na autoria e reconhecimento. Isso requer não apenas um compromisso teórico com a justiça e a equidade, mas também a aplicação prática de diretrizes e práticas que promovam esses ideais em todo o processo de pesquisa colaborativa.

Construir e manter parcerias equitativas requer reflexão contínua e um compromisso com a melhoria contínua, garantindo que todas as vozes sejam ouvidas e valorizadas, e que os benefícios da pesquisa sejam compartilhados de maneira justa e equitativa. É um desafio complexo, mas crucial para o avanço da pesquisa ser não apenas inovador, mas também responsável, inclusivo e, além disso, para que seu progresso transcenda os pesquisadores envolvidos e envolva suas instituições localmente e, por meio delas e de sua liderança e influência, outras instâncias nacionais, regionais e globais sejam impactadas, em um movimento dinâmico e transformador da realidade.

É necessário uma avaliação constante e dedicação ao desenvolvimento contínuo para criar e sustentar colaborações justas e equitativas, garantindo que todas as opiniões sejam ouvidas, que ninguém seja deixado para trás, e que a cultura e anseio de todos sejam respeitadas, proporcionando que as vantagens da pesquisa sejam distribuídas igualmente. Embora pareça uma tarefa difícil, é essencial que a pesquisa seja inovadora, responsável, inclusiva e que seu avanço ultrapasse os pesquisadores individuais e suas instituições localmente, permitindo que outras instâncias nacionais, regionais e globais serão impactadas em um movimento dinâmico e transformador da realidade.

Colaborações em pesquisa justas e equitativas enfrentam muitos obstáculos complexos, especialmente quando se consideram as relações entre instituições do Norte e Sul Global. Institutos de pesquisa de países ricos frequentemente detêm uma quantidade desproporcional de recursos em colaborações para pesquisa global de saúde. A experiência do Brasil durante a pandemia de COVID-19 demonstra amplamente essa dinâmica, especialmente diante das restrições financeiras do país para pesquisa neste momento crucial^(5)^.

O Brasil e várias outras nações de renda média viram reduções significativas em seus orçamentos de pesquisa durante a epidemia. Isso teve um efeito direto na capacidade do país de lidar com a emergência de saúde, realizar pesquisas independentes sobre o vírus, tratamentos e vacinas, e contribuir de forma independente para a compreensão mundial da pandemia. Essa disparidade pode perpetuar uma dinâmica neocolonialista já observada em outros contexto em que nações mais ricas definem a agenda de pesquisa, ignorando a voz, a representativa e a independência das instituições em países em desenvolvimento. Portanto, para avançar na equidade, é imperativo que a comunidade internacional adote medidas concretas anti-racistas e anti-coloniais. Pesquisadores e membros da equipe de nações subdesenvolvidas devem ser permitidas de desempenhar um papel mais proativo e decisivo nas colaborações, desde a identificação de problemas locais, metas e soluções até a negociação das condições de parcerias internacionais nas quais estão envolvidas.

### O impacto de parcerias justas e equitativas nos ODS

As parcerias de pesquisa justas e equitativas têm um impacto profundo e multifacetado na realização dos ODS estabelecidos para 2030. A colaboração entre países de alta renda e países de renda média e baixa em termos de justiça e equidade não é apenas um imperativo ético, mas também uma estratégia eficaz para acelerar o progresso global em direção a esses objetivos. Ao analisar as perspectivas e conclusões apresentadas na literatura, podemos obter uma melhor compreensão da influência dessas parcerias nos ODS^(6)^.

- Fortalecimento da Capacidade de Pesquisa Local (ODS 9 - Indústria, Inovação e Infraestrutura): Parcerias justas e equitativas contribuem para o desenvolvimento de infraestruturas de pesquisa robustas em países em desenvolvimento, fomentando a inovação local e permitindo que esses países não apenas participem, mas também liderem esforços de pesquisa que abordem suas necessidades específicas. Isso não apenas melhora a capacidade de pesquisa local, mas também promove a auto-suficiência e reduz a dependência de intervenções externas.
- Incorporação de Múltiplas Perspectivas (ODS 5 - Igualdade de Gênero, ODS 10 - Redução das Desigualdades): Parcerias equitativas garantem que uma ampla gama de vozes e perspectivas seja incluída na pesquisa, o que é vital para abordar questões de gênero e desigualdade. A inclusão de grupos marginalizados e sub-representados nos processos de pesquisa garante que suas necessidades e desafios sejam adequadamente compreendidos e abordados.
- Promoção da Educação de Qualidade (ODS 4): Ao envolver instituições de países em desenvolvimento em parcerias equitativas, a capacidade educacional e de treinamento nessas regiões é aprimorada. Isso leva a um aprimoramento na qualidade da educação, por meio do estabelecimento de programas de treinamento e capacitação pertinentes às necessidades locais e alinhados com as melhores práticas globais.
- Enfrentamento das Desigualdades em Saúde (ODS 3 - Saúde e Bem-Estar): Parcerias justas e equitativas no campo da saúde global são cruciais para abordar as desigualdades em saúde. Ao colaborar em igualdade de condições, países e instituições podem desenvolver e implementar soluções de saúde culturalmente apropriadas, economicamente viáveis e acessíveis para todas as populações, especialmente as mais vulneráveis.
- Aceleração da Implementação e Impacto dos (ODS 17 - Parcerias para os Objetivos): Parcerias justas e equitativas são exemplos do ODS 17, que enfatiza a importância das parcerias na realização dos ODS. Elas ilustram como a colaboração baseada em respeito mútuo, igualdade e compartilhamento de benefícios pode acelerar o progresso em direção a todos os objetivos.

### Parcerias justas e equitativas em pesquisa são o caminho para 2030

As parcerias de pesquisa justas e equitativas desempenham um papel crítico na consecução da Agenda 2030 para o Desenvolvimento Sustentável. Devido à sua interconexão, elas requerem colaboração entre países, setores e disciplinas. Parcerias de pesquisa, quando conduzidas de maneira justa e equitativa, podem acelerar esse processo de diversas maneiras significativas.

Parcerias justas e equitativas facilitam a co-criação de conhecimento, combinando expertise local e global para abordar questões sensíveis e desafiadoras. A abordagem interdisciplinar e multicultural proporcionada é crucial para compreender e enfrentar os desafios multifacetados que os ODS buscam enfrentar. A co-criação de conhecimento também garante que as soluções sejam culturalmente apropriadas, socialmente aceitáveis e tecnicamente viáveis^(7)^.

As parcerias podem aprimorar as capacidades institucionais e individuais em países de renda média e baixa. Compartilhando conhecimento, recursos e tecnologias, instituições de países de alta renda podem contribuir para o desenvolvimento de infraestruturas de pesquisa sustentáveis, adaptadas às realidades e necessidades específicas dos países em desenvolvimento. Isso não apenas apoia a pesquisa local, mas também contribui para o desenvolvimento de profissionais qualificados e instituições robustas, que são essenciais para o progresso sustentável^(8)^.

Parcerias equitativas enfrentam diretamente as desigualdades globais ao buscar promover um equilíbrio mais justo de poder, recursos e reconhecimento. Ao fazer isso, elas contribuem para alcançar os ODS relacionados à redução das desigualdades (ODS 10) e à promoção de sociedades pacíficas e inclusivas para o desenvolvimento sustentável (ODS 16).

Por meio de parcerias que valorizam as perspectivas locais é possível que os objetivos e estratégias globais estejam alinhados com as necessidades e prioridades locais. Essa abordagem descentralizada é crucial para o sucesso dos ODS, pois reconhece que soluções eficazes devem estar enraizadas no contexto local.

Parcerias equitativas podem apoiar a pesquisa e o desenvolvimento de soluções que promovam a sustentabilidade ambiental (ODS 13, 14 e 15) e garantam o bem-estar social e econômico (ODS 1, 2, 3, etc.). Ao combinar diferentes tipos de conhecimento e recursos, essas parcerias podem inovar em áreas como saúde, educação, agricultura sustentável e energia limpa.

Parcerias de pesquisa justas e equitativas podem, ainda, promover a paz e a justiça ao abordar as raízes estruturais subjacentes das desigualdades e promover sociedades inclusivas e justas, conforme delineado no ODS 16.

### O Papel da Enfermagem em Parcerias de Pesquisa Justas e Equitativas

A Enfermagem, tanto como ciência quanto como prática, desempenha um papel crucial no contexto de parcerias de pesquisa justas e equitativas, especialmente dentro do quadro dos ODS. A orientação holística da profissão - centrada no paciente, aliada ao seu compromisso com o cuidado culturalmente competente, coloca-a em uma posição única para influenciar e liderar parcerias de pesquisa com o objetivo de alcançar os ODS^(9)^. Existem diversas maneiras pelas quais a enfermagem pode se posicionar e contribuir de maneira significativa:

- Advocacia pela Equidade em Saúde: A enfermagem, enquanto posição de liderança, pode advogar pela equidade em saúde trabalhando para garantir que as vozes das comunidades sub-representadas sejam ouvidas em parcerias de pesquisa. Os enfermeiros frequentemente estão na linha de frente do cuidado, o que os coloca em uma posição única para entender as necessidades das comunidades e advogar pela inclusão dessas perspectivas na agenda de pesquisa.
- Pesquisa de base comunitária: Os enfermeiros podem liderar e participar de pesquisas baseadas na comunidade, garantindo que as questões de pesquisa sejam relevantes para as necessidades locais e que os resultados da pesquisa sejam traduzidos em práticas que beneficiem diretamente as comunidades atendidas.
- Treinamento e Educação: A enfermagem pode desempenhar um papel crucial no empoderamento e educação, tanto em nível local quanto global. Os programas de formação em enfermagem podem incluir componentes que enfatizem a importância de parcerias de pesquisa justas e equitativas, preparando assim a próxima geração de enfermeiros para serem colaboradores eficazes e conscientes em pesquisas globais.
- Liderança em Interdisciplinaridade: Devido à sua natureza interdisciplinar e posicionamento central nos sistemas de saúde, a enfermagem está bem posicionada para liderar ou participar de equipes multidisciplinares de pesquisa. Isso pode promover uma abordagem mais holística à pesquisa, garantindo que múltiplas perspectivas sejam incluídas e que os resultados sejam mais sólidos e aplicáveis, desde que ela reivindique assento à mesas decisórias e compareça com sua expertise oferecendo sugestões, planos e estratégias de implementação
- Advocacia por Políticas Baseadas em Evidências: Os enfermeiros, como defensores de políticas baseadas em evidências, podem utilizar os resultados da pesquisa para influenciar e moldar políticas de saúde que promovam equidade, justiça e desenvolvimento sustentável. Para assim poder agir, os enfermeiros precisam conquistar espaços em que as políticas são estabelecidas, estar atualizado em termos das evidências produzidas pela enfermagem, para então liderar e apresentar propostas fundamentadas exercendo sua influência no estabelecimento de políticas.
- Através de parcerias globais, os enfermeiros podem colaborar com colegas em diferentes regiões para compartilhar conhecimento, recursos e melhores práticas. Isso não apenas melhora as capacidades locais, mas também promove uma compreensão mais profunda dos determinantes sociais da saúde em contextos culturais e geográficos diversos.

Assumindo os papéis de líderes, educadores, pesquisadores e defensores, os enfermeiros podem desempenhar um papel crucial em garantir que as parcerias de pesquisa sejam justas, equitativas e alinhadas com os objetivos destinados a promover a saúde global e o desenvolvimento sustentável. A enfermagem, com seu compromisso inabalável com o cuidado, empatia, afirmação dos direitos humanos justiça social, tem um papel significativo a desempenhar na conquista dos por meio de parcerias de pesquisa fortes e colaborativas^(10)^.

## CONSIDERAÇÕES FINAIS

Nosso estudo enfatiza a necessidade crítica de parcerias de pesquisa justas e equitativas no caminho para alcançar os Objetivos de Desenvolvimento Sustentável (ODS) até 2030. Ao reconhecer a diversidade de perspectivas, conhecimentos e recursos que diferentes instituições e indivíduos trazem para o processo de pesquisa, podemos promover uma abordagem mais holística e inclusiva para enfrentar os desafios globais de saúde e desenvolvimento. Ao fortalecer as capacidades locais, incorporar outras perspectivas e promover equidade em saúde, as parcerias de pesquisa podem desempenhar um papel crucial na transformação de nossas sociedades em direção a um futuro mais justo e sustentável.

Além disso, neste estudo destacamos o papel crucial da enfermagem como protagonista na promoção de parcerias de pesquisa equitativas e na busca pela realização dos ODS. A profissão de enfermagem, com sua abordagem holística e centrada no paciente, está bem posicionada para liderar iniciativas de pesquisa comunitária, advogar por políticas baseadas em evidências e fortalecer as capacidades locais de saúde. Ao integrar ativamente a enfermagem em parcerias de pesquisa, podemos garantir que as soluções sejam culturalmente sensíveis, socialmente equitativas e clinicamente eficazes, impulsionando assim o progresso em direção aos ODS.
